# Microwave-Based Oxidation State and Soot Loading Determination on Gasoline Particulate Filters with Three-Way Catalyst Coating for Homogenously Operated Gasoline Engines

**DOI:** 10.3390/s150921971

**Published:** 2015-09-02

**Authors:** Markus Dietrich, Christoph Jahn, Peter Lanzerath, Ralf Moos

**Affiliations:** 1 Department of Functional Materials, Bayreuth Engine Research Center (BERC), Zentrum für Energietechnik (ZET), University of Bayreuth, 95440 Bayreuth, Germany; E-Mails: Functional.Materials@uni-bayreuth.de (C.J.); Functional.Materials@uni-bayreuth.de (R.M.); 2 Daimler AG, 70546 Stuttgart, Germany; E-Mail: peter.lanzerath@daimler.com

**Keywords:** gasoline particulate filter (GPF), three-way catalytic converter (TWC), oxygen storage capacity (OSC), on-board diagnostics (OBD), microwave cavity perturbation method, New European Driving Cycle (NEDC), lambda probe

## Abstract

Recently, a novel method emerged to determine the oxygen storage degree of three way catalysts (TWC) by a microwave-based method. Up to now, this method has been investigated only in lab-scale reactors or under steady state conditions. This work expands those initial studies. A TWC-coated gasoline particulate filter was investigated in a dynamic engine test bench simulating a typical European driving cycle (NEDC). It could be shown that both the oxygen storage degree and the soot loading can be monitored directly, but not simultaneously due to their competitive effects. Under normal driving conditions, no soot accumulation was observed, related to the low raw emissions and the catalytic coating of the filter. For the first time, the quality factor of the cavity resonator in addition to the resonance frequency was used, with the benefit of less cross sensitivity to inconstant temperature and water. Therefore, a temperature dependent calibration of the microwave signal was created and applied to monitor the oxidation state in transient driving cycles. The microwave measurement mirrors the oxidation state determined by lambda probes and can be highly beneficial in start-stop phases (where lambda-probes do not work) and to determine the oxygen storage capacity (OSC) without unnecessary emissions.

## 1. Introduction

The exhausts of homogeneously operated gasoline engines are commonly cleaned by three-way catalytic converters (TWCs) [1]. For maximum conversion of the limited components hydrocarbons (HC), carbon monoxide (CO), and nitrogen oxides (NO_x_), the engine is operated stoichiometrically (air-fuel ratio λ = 1). To buffer lean-rich variations, the washcoat contains doped ceria as an oxygen storage component [2]. Under lean conditions (λ > 1), ceria is oxidized to CeO_2_, whereas under rich conditions (λ < 1), it is reduced to Ce_2_O_3_. Today, TWCs are monitored and controlled indirectly by lambda probes up- and downstream of the catalyst [1,3,4]. The oxygen storage capacity (OSC) of the washcoat is highly temperature dependent and decreases with aging. Today’s vehicles determine the OSC indirectly by models relying on the lambda probe signals [5].

Besides gaseous components, the more stringent upcoming emission standards may force the automotive industry to reduce particulate matter emissions as well, even for gasoline engines. Gasoline particulate filters (GPF) have been suggested for this purpose [6,7]. They are similar to diesel particulate filters (DPF), which are already common for diesel engines. Soot and ash are continuously trapped in ceramic wall-flow filters. From time to time, soot has to be regenerated thermally to avoid clogging. For filter loading state determination, models are used involving the pressure drop over the filter [8,9]. When transferring the DPF concept to gasoline engines, filters with a TWC coating are appropriate to avoid unnecessary additional parts in the exhaust pipe [10]. Coated filters are already in use for combined systems of selective catalytic reduction applications (SCR-DPF) [11,12] or to enhance soot oxidation at low temperature [13].

In order to determine *in-situ* and during operation the oxidation state of a TWCs or the soot loading in DPFs, the microwave cavity perturbation method has been suggested [14,15,16,17,18]. It uses the metal canning of the catalyst to form an electrical cavity resonator. The dominant effect in this technique is the change of the dielectric permittivity of the cavity filling, as the catalyst stores oxygen, or the filter accumulates soot. It is known that the conductivity of ceria changes by several decades upon reduction or oxidation [19,20,21,22] and in several studies, it could be shown that the observed microwave data correlate well to the oxygen storage of a TWC and is neither cross sensitive to CO_2_ or H_2_O nor dependent on the gas flow [15,23,24,25,26]. The increasing dielectric losses, stemming from the conductive soot particles, are assumed to be the main effect of soot loading [17,18,27]. Interestingly, other serial devices in the exhaust gas aftertreatment can also be determined by this technique. The storage degree of nitrogen monoxide (NO) in lean NO_x_ traps [28,29] and the ammonia loading of SCR catalyst devices have also been successfully monitored [30,31].

In this paper, we present initial studies with microwave cavity perturbation technique on a gasoline particulate filter (GPF) with TWC catalytic coating for homogeneously operated gasoline engines. For the first time ever, the microwave state observation was performed for TWC coatings, using transient standardized driving cycles, including start-stop phases. Furthermore, it has never been shown before that both antennas can be placed in the (soot-free) downstream side of the filter/catalyst.

## 2. Microwave Cavity Perturbation for Catalyst and Filter State Determination

The microwave cavity perturbation technique uses standing electromagnetic waves (resonances) inside a defined metal canning. The presence of a volume with a different permittivity than the remaining cavity filling leads to a perturbation of the electromagnetic standing waves, visible in a decrease in resonance frequency and in an increase of the 3 dB bandwidth of the resonance peak (*i.e.*, in a decrease of the quality factor, *Q*). In the following, we first show the idealized theory with the basic effects for high-quality cavity resonators for material characterization, then we extrapolate this behavior to the special case of a cavity with a sample that occupies most of the resonator volume as it is applied in typical exhaust gas aftertreatment devices.

To be sensitive to permittivity changes, the sample has to be placed in regions with high electrical field strength. In material characterization, a very small sample (compared to the cavity volume) is used and placed in the electrical field maximum. Then, Equations (1) and (2) are valid to calculate the complex dielectric permittivity ε = ε_1_ − jε_2_ of the sample. The real part ε_1_ (or more properly, ε_1_ − 1), as a measure of polarization, is connected to the change in resonance frequency from *f*_0_ to *f_s_*. To determine ε_2_, quantifying the dielectric losses, the so-called 3 dB bandwidth *BW* of the resonance peak is required, represented by the change of the unloaded quality factor from *Q*_0_ to *Q_s_*. For a symmetrically coupled cavity, the unloaded quality factor (without coupling influences) can be calculated by Equation (3), requiring the resonance frequency *f*, the 3 dB bandwidth *BW* and the resonance peak height |*S*_21, max_| (the extraction of the three values from a resonance peak is schematically shown in the experimental section). Additionally, the volume of the sample *V_s_* and the effective volume of the resonator *V_eff_* (integration of the electrical field distribution over the cavity volume, depending on the analyzed resonance) is required [32]:

(1) $$\frac{f_{0}-f_{s}}{f_{0}}\approx \left(\varepsilon_{1}-1\right)\frac{V_{s}}{2V_{eff}}$$

(2) $$\frac{1}{Q_{s}}-\frac{1}{Q_{0}}\approx \varepsilon_{2}\frac{V_{s}}{V_{eff}}$$

(3) $$Q=\frac{f}{BW}\left(1-10^{-|S_{21,\max}|/20}\right)$$

In the application-relevant case, at which the sample occupies most of the cavity volume, the electrical field strength varies over the sample length and the shown Equations (1) and (2) are obviously not valid. Nevertheless, the basic response of the resonance to changes in polarization and losses of its filling is still the same as for the ideal cavity. Therefore, the simplified Equations (4) and (5) with the unknown dimensionless constants *A* and *B* can be introduced, since the desired pieces of information are the catalysts’ states and not their exact dielectric properties. For application, the knowledge of defined catalyst states is necessary to calibrate the catalyst state observation system:

(4) $$\frac{\Delta f}{f}\approx A\left(\varepsilon_{1}-1\right)$$

(5) $$\Delta \left(\frac{1}{Q}\right)\approx B\;\varepsilon_{2}$$

For material characterization cavities it is common to use weak coupling elements. Owing to the big sample volume and the high losses, the catalyst cavity requires a stronger coupling (overcoupling). If not, the resonances would be close to the recording limit of the network analyzer. Additionally, the harsh atmosphere and the resulting stress inside of an exhaust pipe require robust coupling elements.

## 3. Experimental

**Figure 1:**
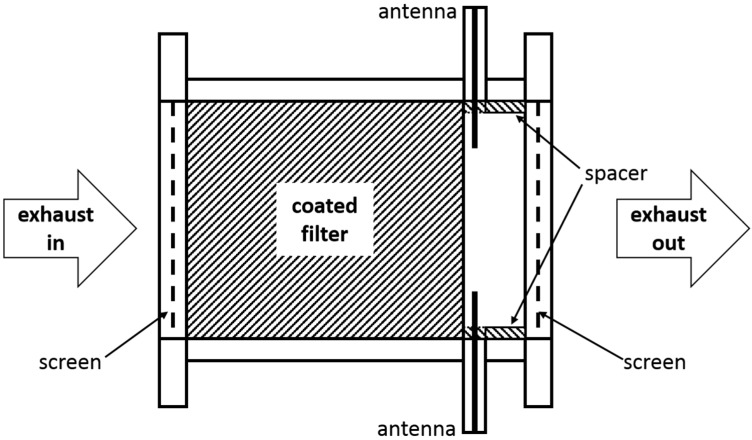
Illustration of the filter canning, used for the engine experiments: for the microwave measurements, two probe antennas downstream of the GPF and two metal screens (one up- and one downstream of the GPF) were added to the conventional system.

The samples under investigation were two TWC-coated particulate filters on cordierite substrates with the identical content of catalytic active materials, only differing in the ceria content of the washcoat. The first filter “**C1**” has a comparable ceria content as a common TWC, the second filter “**C2**” has double the ceria content. Gas and soot loading experiments were performed on an engine test bench with a homogenously operated and turbocharged 6-cylinder gasoline engine with a twin pipe exhaust system. Both coated filters (diameter of 4.66″ and length of 4.5″) were placed successively in closed coupled position instead of a conventional TWC. Figure 1 schematically illustrates the modified canning for the microwave measurements. The filters were pressed into the metal canning with two microwave probe antennas to measure in transmission mode. Both antennas were placed axially opposed on the downstream side to avoid disturbing soot influences. A spacer ring was provided to ensure the filters in a fixed position and to protect the antennas. For a defined cylindrical cavity shape, two metal screens were flanged up- and downstream of the canning.

To investigate the influences of gas and soot loading on the coated filters, several types of test cycles were necessary. For normal driving conditions including city traffic with start-strop phases and highway driving, the common New European Driving Cycle (NEDC 2000, test length 11 km, test time 1180 s, maximum speed 120 km/h) was applied, with both cold and warm starts. In addition, a special OSC test cycle to determine the oxygen storage capacity of the filters with continuous lean-rich changes at five different operation points was used. Under normal operation conditions, the used engine does not emit much particulate matter. In order to test strong soot loadings on the filters in a short time, the engine was operated permanently rich with additional fuel injections; a deliberately selected, fully non-realistic operation with a high soot emission. Besides the microwave system, with the two coaxial capacitive probe antennas, two 50 Ω coaxial cables, and the network analyzer (MS2028B, Anritsu, Morgan Hill, CA, USA), the standard measurement setup, including thermocouples, lambda probes up- and down-stream of the filters and differential pressure sensors were logged.

**Figure 2:**
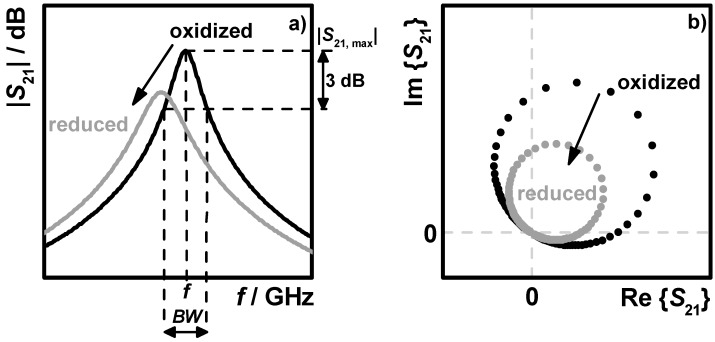
Schematic illustration of spectra of the complex transmission parameter *S*_21_ with catalyst in the oxidized (black) and reduced (grey) state: (**a**) magnitude with resonance peak and resonance parameters *f*, *BW* and |*S*_21, max_| for the oxidized state; and (**b**) plot in the complex plane with resonance circle.

The complex transmission parameter *S*_21_ was recorded with an acquisition rate of 1 Hz between 1.2 and 1.7 GHz. This frequency range included the lowest appearing resonance mode (TE_111_) with one electric field maximum and no zero field regions over the filters. Schematic illustrations of the transmission parameter are displayed in Figure 2, in a fully oxidized (black) and a fully reduced state (grey). Figure 2a shows the logarithmically plotted data with the shifting resonance peak and the resonance parameters *f*, *BW* and |*S*_21, max_| for the oxidized state, and Figure 2b shows the corresponding complex data with resonance circles, changing in size and position. To determine the resonance frequency and the unloaded quality factor (Equations (4) and (5)), a complex fitting approach has been used. By circle fitting and transforming the resonance circles into the so-called canonical position, the phase-frequency relation let to an accurate resonance frequency and quality factor determination [33,34]. Related to Equations (4) and (5), the two considered measurement values corresponding to the changes of ε_1_ and ε_2_ are the resonance frequency *f*_res_ and the reciprocal unloaded quality factor *Q*_0_^−1^. For the case of very high loss (resulting from high soot loading), the determination of the resonance frequency and the quality factor was not possible. Then the average magnitude value of the recorded transmission parameter *S*_21_ has been used, similarly as reported in [35].

## 4. Results and Discussion

### 4.1. Oxidation State Determination under Transient Conditions

In former work [15,20,24,26], the oxidation state determination was conducted under controlled and constant catalyst temperatures. For transient conditions, there are two main effects influencing the cavity perturbation measurements related to temperature. Firstly, there is a geometry change resulting from thermal expansion of the metal canning. This increases the cavity and leads to a lower resonance frequency. Secondly, the material properties of the coated filter depend on temperature. Both, the oxygen storage state (which is the intended measurand) and the conductivity of the coating (which is the physical quantity that is deduced from the measured microwave signal) have a strong temperature dependency, both influencing the resonance frequency and the quality factor. With respect to the non-constant exhaust gas and catalyst temperatures during real-world applications, a temperature compensation is necessary. For TWC coatings, the material effects usually dominate the changes of the resonance frequency, but for other catalyst types with smaller dielectric changes, both effects may be equally large.

**Figure 3:**
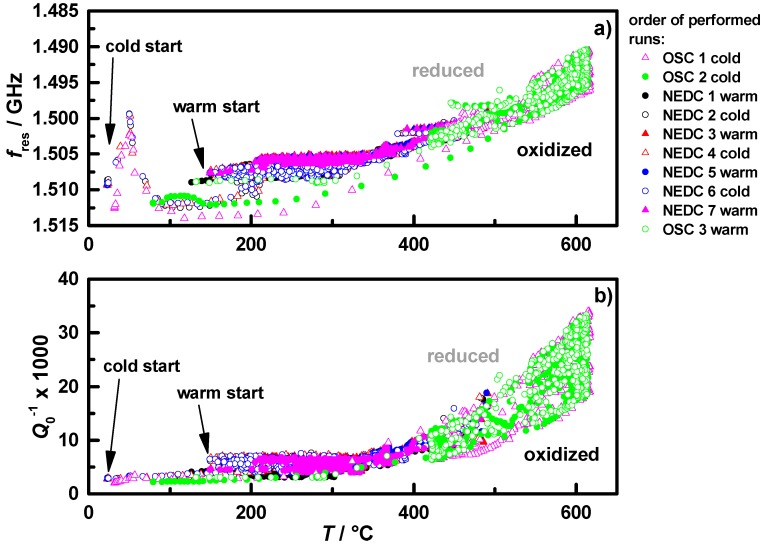
Temperature dependence of (**a**) *f*_res_ in reverse scale and (**b**) *Q*_0_^−1^, observed during NEDC (max. 500 °C) and OSC runs (max. 620 °C) under cold and warm start conditions for sample **C1**; each differently colored data set represents one experimental run.

Figure 3 shows the measured resonance frequency in reversed scale (a) and the reciprocal unloaded quality factor (b) as a function of the measured gas temperature downstream of all conducted NEDC and OSC runs with cold and warm start conditions of the coated filter **C1**. Each colored symbol type represents one test cycle. The lower temperature region from 25 to 500 °C is mostly represented by the NEDC and the higher temperatures up to 600 °C by the OSC measurements. Based on the theory, *f*_res_ shifts to a lower frequency and *Q*_0_^−1^ increases with increasing complex permittivity of the cavity filling. In the performed transient measurements, the coated filter reached different oxidation states at various temperatures. All data points together offer a characteristic map of the oxidation state as a function of temperature with the upper border representing the fully reduced state and the lower border representing the fully oxidized state of the catalytically coated filters. Between 120 and 300 °C, both, *f*_res_ and *Q*_0_^−1^, form almost horizontal borders. For temperatures over 300 °C, the values and the differences between fully oxidized and fully reduced increase, an observation that coincides fully with [36].

From Figure 3 it becomes clear that both analyzed values, *f*_res_ and *Q*_0_^−1^, have mainly similar temperature dependencies, but *f*_res_ seems to be additionally influenced by other effects. One of these effects appears to be water, visible in the cold start measurements between 25 and 200 °C, as already observed in lab experiments [36]. In the same temperature region, *Q*_0_^−1^ shows a significantly lower sensitivity to water. This behavior could be explained by the correspondence of *f*_res_ to ε_1_, which is a measure of polarization and the polar nature of water molecules. Additionally, *f*_res_ shows no completely reproducible behavior in the continuously performed measurements, for example visible in the pink points compared to the blue and green data points in Figure 3a. A possible soot accumulation in the NEDCs can be excluded since no change in differential pressure was detected and no irregularity in the changes in *f*_res_ over the test cycles occurred. In contrast, the values of *Q*_0_^−1^ determined in all experimental runs fit together very well and do not show any drift behavior. This again may be explained be the insensitivity of *Q*_0_^−1^ to the thermal expansion of the canning and by the small influence of water to the losses. The same behavior occurred for the filter with twice the Ceria content **C2**. It appears that *Q*_0_^−1^ is the more appropriate measurand for the oxidation state determination of the TWC coated GPF compared to the resonance frequency, *f*_res_. Hence, we will focus in the following on *Q*_0_^−1^ to determine the oxidation state during engine operation.

**Figure 4:**
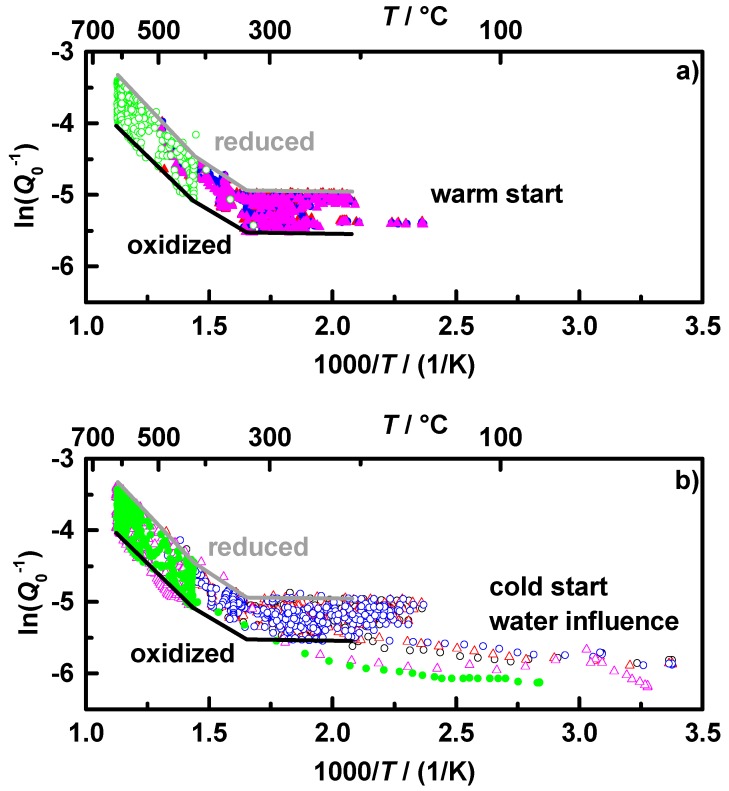
Arrhenius-like representation of *Q*_0_^−1^ of the conducted NEDC and OSC runs with sample **C1** according to Figure 3b: (**a**) warm start and (**b**) cold start conditions, with the characteristic curve of the oxidized (black) and reduced (grey) state; each differently colored data set represents one experimental run. The same labels as shown in Figure 3 apply.

According to Equation (5), *Q*_0_^−1^ is proportional to the change of ε_2_, which itself is proportional to the conductivity for semiconducting materials in the GHz range by ε_2_ = *σ*/(2π*f*·ε_0_) [37]. Hence, the change in *Q*_0_^−1^ can be seen as a direct measure of a change in conductivity of the filter coating. As seen in Figure 3b, *Q*_0_^−1^ appears to be thermally activated. Therefore, the assumption of an Arrhenius-like behavior of *Q*_0_^−1^ for a characteristic map of the temperature dependency of the oxidation state is appropriate. Figure 4a shows the Arrhenius plot of the warm start and Figure 4b the cold start measurements of the filter **C1**. According to the data points of the warm start measurements in Figure 4a, characteristic lines were fit to the borders for the reduced (grey) and oxidized state (black). It is clearly visible that the data fit has three sections with three different slopes for each state. The temperatures at which the slopes change seem to be the same for the oxidized and the reduced state. The characteristic lines created by the warm start measurements marked into the cold start measurements in Figure 4b fit very well. However, it seems to be necessary that the exhaust gas (not the TWC coated GPF itself!) has reached temperatures above 200 °C to show negligible water influence. In order to use the created map for oxidation state determination in the NEDCs, the minimum downstream gas temperature for this analysis was set to 150 °C, with assuming almost 200 °C in the coated filter.

**Figure 5:**
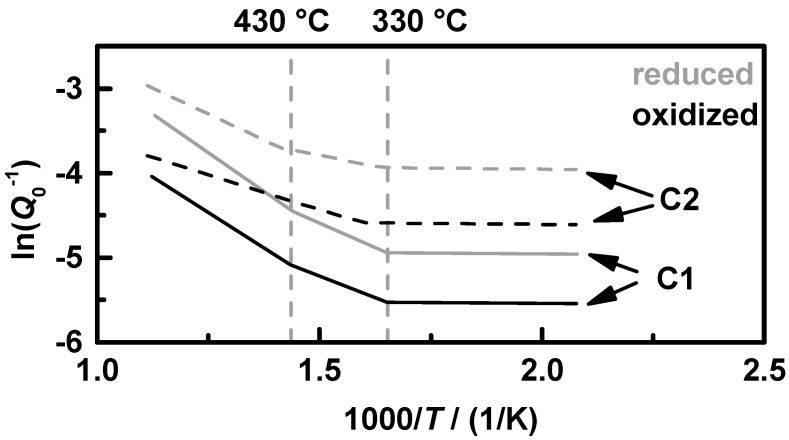
Comparison of the characteristic curves of *Q*_0_^−1^ in an Arrhenius-like representation: the oxidized (black) and reduced (grey) state for the samples **C1** (solid) and **C2** (dashed) with doubled Ceria content.

Before using the characteristic map to analyze the current oxidation state in a test cycle, we will have a short comparison of the two tested coated filters. The above-described procedure for filter **C1** has also been performed for filter **C2**. The fitted characteristic oxidation state lines for both filters are displayed in Figure 5: oxidized (black) and reduced (grey) for **C1** (solid) and **C2** (dashed). Both filters show a very similar behavior. The filter **C2** has always a higher value of *Q*_0_^−1^ than **C1**, according to its higher ceria content and the resulting higher conductivity. The two changes in the slopes are for both samples at almost the same temperatures, at 330 and 430 °C. It seems that the filter coating has three areas with different thermal activation energies. However, resulting of the fact that *Q*_0_^−1^ has an unknown proportionality to the conductivity, these results do not allow a statement about values of activation energies or even a comparison to other materials. At this point, a recently in [38] introduced setup using the cavity perturbation method for an exact material characterization of catalysts could be helpful for further investigations of the dielectric properties and conductivity mechanisms of TWC coatings in the GHz range.

**Figure 6:**
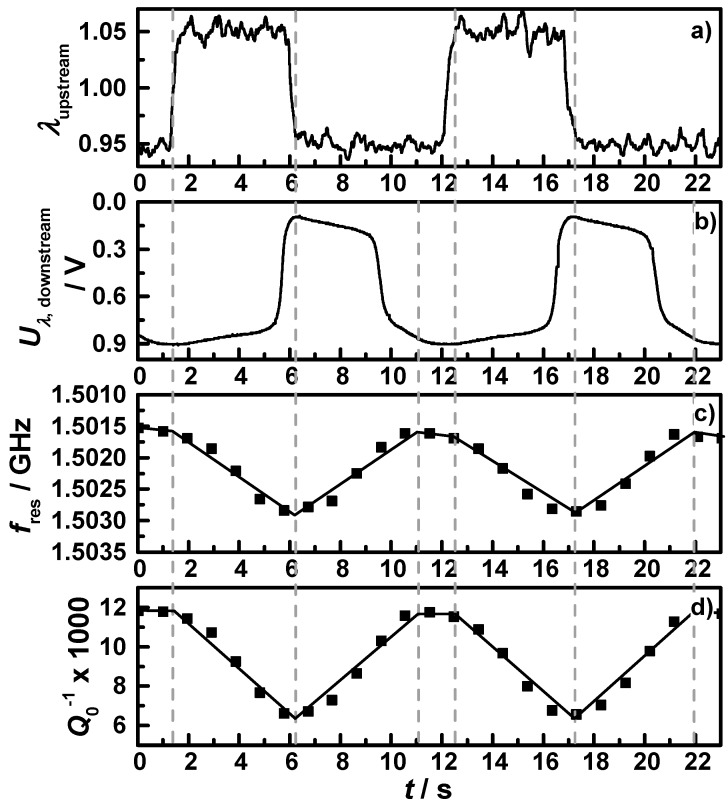
Section of a performed OSC measurement of filter **C1** with continuous lean-rich changes at 425 °C: (**a**) the air-fuel ratio λ measured by the upstream broadband lambda probe; (**b**) downstream lambda probe signal; (**c**) the resonance frequency in reverse scale; and (**d**) the reciprocal quality factor.

Besides the fully oxidized and reduced state, the knowledge about the behavior in the intermediate oxidation states is necessary. Former work already showed a linear relation between the oxidation state and the resonance frequency [24]. To investigate the relation between oxidation state and microwave data, Figure 6 shows exemplarily a short section of one OSC measurement at the operation point at 425 °C, with (a) the air-fuel ratio λ as derived by the upstream broadband lambda probe; (b) the downstream lambda probe signal; (c) the resonance frequency in reverse scale; and (d) the reciprocal quality factor. Vertical grey dashed lines indicate the point in time when either the upstream broadband lambda probe or the downstream lambda probe switched from lean to rich or *vice versa*. According to the changes of the lambda probe signals and the measured data points, black lines are drawn into the two microwave signals representing the idealized signal that would mirror the oxidation state of the coated filter. One can clearly see that the assumption of an almost linear relation between the oxidation state and both the resonance frequency and the reciprocal quality factor is appropriate. The small changes in the slope and the resulting deviations between the drawn lines and the measured data points are related to the locally distributed electric field strength of the TE_111_ mode, which has a maximum in the middle of the cavity and decreases to the front and the end of the catalyst. Nevertheless, it correlates well and this relation was observed in all conducted OSC measurements at all operation points. Since we focused on the quality factor, the oxidation state can be calculated with the knowledge of the fully oxidized and reduced state with Equation (6) for transient conditions with an estimated maximum error less than 10%:

(6) $$O_{2}\;storage=1-\frac{Q_{0}^{-1}-Q_{ox}^{-1}\left(T\right)}{Q_{red}^{-1}\left(T\right)-Q_{ox}^{-1}\left(T\right)}$$

**Figure 7:**
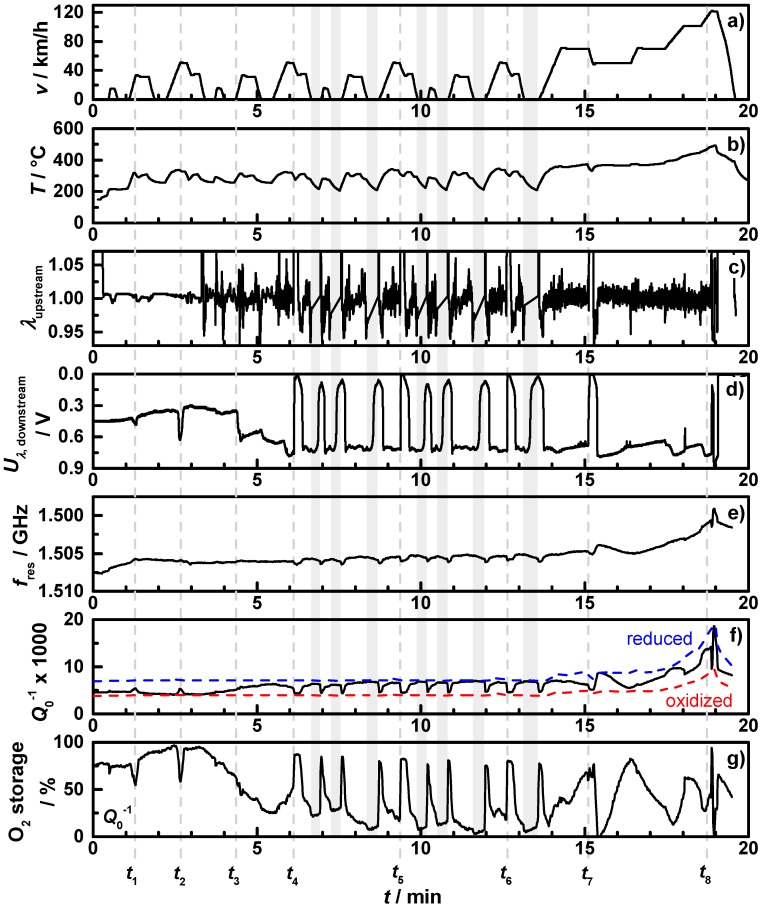
NEDC measurement with warm start conditions for sample **C1**: (**a**) vehicle speed; (**b**) downstream exhaust gas temperature; (**c**) the air-fuel ratio λ measured by the upstream broadband lambda probe; (**d**) downstream lambda probe signal; (**e**) resonance frequency in reverse scale; (**f**) reciprocal quality factor with characteristic lines for temperature dependent reduced (blue) and oxidized (red) state and (**g**) the oxygen storage degree calculated by the quality factor and the characteristic lines. Start-stop phases with engine shut off are shaded grey.

The application of the characteristic map of the oxidation state of Figure 4 and Figure 5 for filter **C1** on an NEDC with warm start conditions is displayed in Figure 7. It shows (a) the theoretical vehicle speed; (b) the downstream exhaust gas temperature; (c) the air-fuel ratio λ measured by the upstream broadband lambda probe; (d) the downstream lambda probe signal; (e) the resonance frequency in reverse scale; (f) the reciprocal unloaded quality factor with the lines for the exhaust gas temperature dependent oxidized (*Q*_ox_^−1^(*T*), red dashed) and reduced state (*Q*_red_^−1^(*T*), blue dashed); and (g) the calculated oxygen storage degree, using Equation (6).

The start-stop phases with engine shut-off are marked by grey shades. The NEDC is divided into urban cycles with start stop intervals (until minute 13.5) and an extra-urban cycle with highway speed. The additionally displayed resonance frequency and the reciprocal quality factor have a similar behavior, but *f*_res_ appears less affected by the changing catalyst state.

After the start, the upstream lambda probe shows for the first three minutes almost no control activity. In this time span, there are two short accelerations (*t*_1_, *t*_2_) resulting in small downstream lambda drops. In the microwave-derived oxygen storage degree, these two drops are clearly visible. After the third minute, the upstream lambda probe shows more control activity.

**Figure 8:**
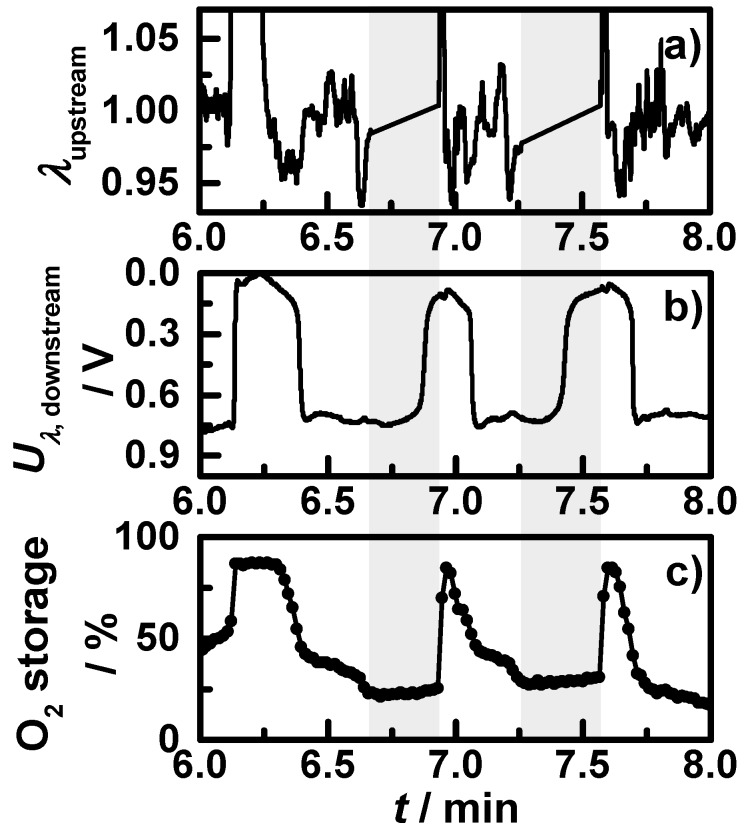
Section of the NEDC measurement with warm start conditions from Figure 7 showing the first two start-stop phases (shaded grey): (**a**) the air-fuel ratio λ measured by the upstream broadband lambda probe; (**b**) downstream lambda probe signal; and (**c**) the microwave-derived oxygen storage degree.

The oxygen storage degree and the downstream lambda signal decrease correspondingly; e.g., the stepwise downstream lambda probe change at *t*_3_ goes along with slowly decreasing oxygen storage degree. At several points in time (*t*_4_, *t*_5_, *t*_6_, *t*_7_), the engine brake is used for deceleration, with the result of a fuel-cut leading to both a temperature drop and a prompt fully oxidation of the catalyst. This is mirrored by both lambda probe signals as well as by the oxygen storage degree. After the sixth minute, the start-stop phases with engine shut-off occur in the cycle. The first two start-stop phases are shown in a close-up view in Figure 8, with (a) the air-fuel ratio λ measured by the upstream broadband lambda probe; (b) the downstream lambda probe signal; and (c) the measured oxygen storage degree. One can see: when the engine is shut off, no air-fuel ratio λ signal can be obtained of the upstream broadband lambda probe and the signal of the downstream lambda probe increases; most likely an effect of oxygen diffusing from the end of the exhaust pipe to the lambda probe. In this time, the measured oxygen storage degree remains on an almost constant level. 

When the engine starts again, the TWC coating gets oxidized by a short lean pulse. In all phases of the urban cycles following to a full oxidation, the oxygen storage degree decreases slowly until the next start-stop or engine brake phase occurs. After the last start-stop interval (around minute 13.5), the last part of the NEDC with the extra-urban cycle starts. From *t*_7_ to *t*_8_, the oxygen storage degree changes continuously with different slopes corresponding to accelerations, always mirrored by the lambda probe signals.

**Figure 9:**
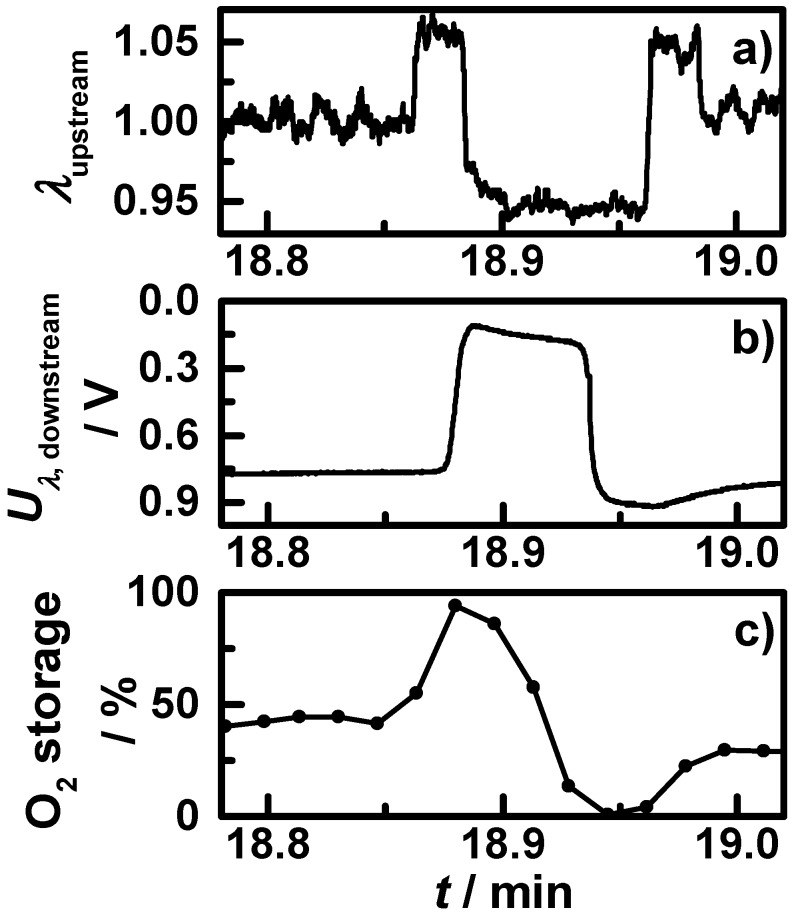
Section of the NEDC measurement with warm start conditions from Figure 7 showing the OSC step: (**a**) the air-fuel ratio λ measured by the upstream broadband lambda probe; (**b**) downstream lambda probe signal; and (**c**) the microwave-derived oxygen storage degree.

In the end of each NEDC (after *t*_8_), the engine control has to test the oxygen storage capacity (OSC) for on-board diagnosis (OBD), by short lean-rich-lean jumps and analyzing the time a full reduction requires. A closer look on this OSC step is displayed in Figure 9, with (a) the upstream broadband lambda probe; (b) the downstream lambda probe and (c) the microwave-derived oxygen storage degree. In the beginning, the upstream air-fuel ratio is hold almost constant around one, then the engine changes to lean conditions until the downstream lambda probe signal indicates a lean atmosphere. Then the TWC coating is fully oxidized. The microwave-derived oxygen storage degree increases corresponding to the lambda probes signals from 40% to 100%. Then the engine control switches to rich conditions until the downstream lambda probe signal indicates rich. The oxygen storage degree decreases and reaches exactly 0%. After a lean pulse and stoichiometric conditions, the oxygen storage degree remains at 30% in the end. The measured oxygen storage degree of the OSC step in the NEDC fits perfectly to the lambda probe signals. This demonstrates that the microwave-based technique is more accurate for the oxygen storage capacity determination than the approach utilizing the downstream lambda probe signal. Additionally, this technique could be able to determine the OSC by analyzing the slope of the oxidation state change without the need to fully oxidize or reduce the catalyst to ensure conversion and avoid unnecessary emissions in the OBD step.

### 4.2. High Soot Loading Experiments

**Figure 10:**
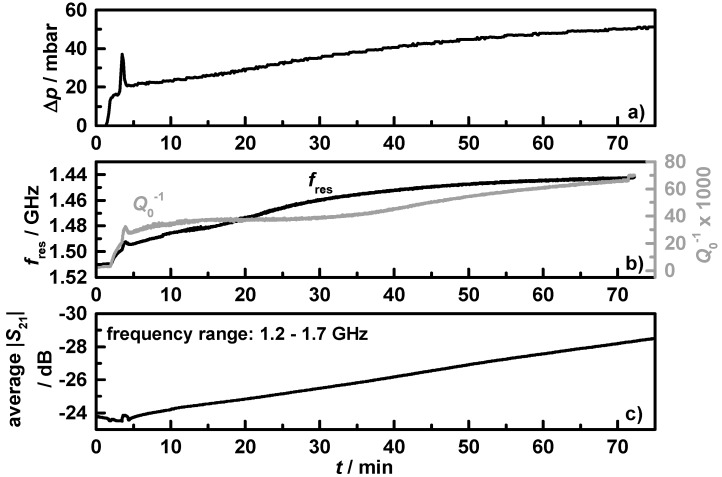
Beginning of the 6 h soot loading experiment on filter **C1** with (**a**) the differential pressure; (**b**) the resonance frequency (black) and reciprocal quality factor (grey); and (**c**) the averaged transmission parameter in the frequency range from 1.2 to 1.7 GHz.

During the conducted NEDCs no notable soot accumulation was observed, neither by the differential pressure sensor nor by the microwave-derived data. Besides the known low raw soot emission of the used homogeneous gasoline engine, the catalytic filter coating could be the reason, since all accumulated soot oxidizes instantaneously under normal driving conditions [7]. To investigate the influence of fully non-realistic high soot loadings, the engine was forced for six hours to produce high amounts of soot and to prevent an unwanted soot oxidation by an operation under constant extreme rich conditions (λ = 0.85). Figure 10 shows the results of the first 75 min of the soot loading experiment for filter **C1**, with (a) the differential pressure, Δ*p*; (b) the resonance frequency (black) and the reciprocal quality factor (grey); and (c) the averaged transmission parameter in the frequency range from 1.2 to 1.7 GHz. Under the used extreme rich conditions, the engine produced high amounts of soot, mirrored in the instantaneous differential pressure increase. In the first minutes of soot loading, Δ*p* increases fast and then slower with a constant slope. This behavior is known from DPF, where the pores inside the ceramic walls get filled first [39]. With filled pores the filter efficiency and the differential pressure is higher and the soot accumulates mostly on and no more in the filter walls. The differential pressure peak around minute four resulted from a short change in operating conditions with higher exhaust gas flow and temperature. Figure 10 gives clear evidence that the resonance frequency and the reciprocal quality factor mirror the differential pressure signal. The already mentioned peak is also visible as an effect of the short increase in temperature in the microwave data (but is much smaller, since the mass flow does not affect the resonance behavior [40]). Due to the high losses of the accumulated soot, the resonance is only analyzable for the first 70 min. Therefore, the averaged transmission parameter was used for higher soot loading determination, and shows an almost linear decrease over the first 75 min from −24 to −29 dB. Such a procedure has been suggested for microwave-based DPF loading estimation by several authors [17,35,40].

**Figure 11:**
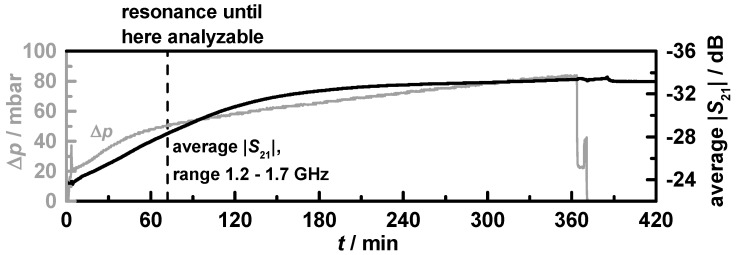
Entire 6 h-lasting soot loading run (filter **C1**). Differential pressure (grey) and averaged transmission parameter in the frequency range from 1.2 to 1.7 GHz (black) agree well. After *ca.* 70 min (black dashed line), the losses were too high and the resonance peak could not be evaluated anymore.

The whole six-hour lasting soot loading run for filter **C1** is shown in Figure 11. The solid grey curve stands for the differential pressure and the averaged transmission parameter is indicated by the black curve. 

The point in time when *f*_res_ and *Q*_0_^−1^ were no appropriate means anymore to measure the soot loading is marked with a black dashed line. It becomes obvious that the averaged transmission, which changes from −24 to −34 dB during the six-hour-lasting loading period, correlates well with the soot loading as indicated by the differential pressure. After the engine was stopped, the signal remained on its level. The filter with doubled Ceria content **C2** showed almost identical results.

Ash loading of filters is an issue which has not been considered here. It is clear, for a long-term investigation, one may expect that ash, which cannot be removed by oxidation, accumulates in the coated GPF and affects the resonance frequency and perhaps also the quality factor. Effects on ash loading have been described recently described for DPF by Sappok and Bromberg [41]. Whether the strategies suggested there can be applied for GPF as well, may be investigated in the future.

## 5. Conclusions and Outlook

For the first time, gasoline particulate filters (GPF) with a TWC coating were analyzed by the microwave-based oxygen and soot loading determination. Additionally, it was shown that such a system gives good results in a transient engine test (NEDC) which includes also start-stop phases. Two filters with different ceria contents (*i.e.*, different oxygen storage capacity) were monitored. By analyzing the resonance frequency, the reciprocal quality factor and the averaged transmission parameter between 1.2 and 1.7 GHz, both oxidation state and soot loading can be monitored separately. This work showed that for oxidation state analysis the reciprocal quality factor appears to be the better parameter instead of the resonance frequency, related to less cross sensitivities especially to water and temperature.

Resulting on the fact that oxidation state and soot loading determination rely on the same measureable effects, a high soot loading prevents oxygen storage degree determination. Nevertheless, the conducted realistic driving cycles showed that under normal driving conditions the here-used gasoline engine emits almost no soot and if so, it does not accumulate in the filter. The latter may be a benefit of the catalytic coating, resulting in an instantaneous oxidation of the soot on the filter washcoat.

A temperature dependent calibration map for the investigated filters to determine the oxidation state under transient conditions was created and used to determine the oxygen storage degree during several New European Driving Cycles. The directly measured, microwave-derived oxygen storage degree correlates well with the indirect lambda probe signals up- and downstream of the coated filters. It is beneficial that—in contrast to the lambda probe-based method—the oxygen storage degree can even be measured in start-stop phases, when the engine is turned off. It is noteworthy that the microwave-based technique allows to determine the oxygen storage capacity (OSC) during the OBD procedure using lean-rich-lean can be more accurately than with the dual lambda probe method and without the need to fully oxidize or reduce the coated filter or catalyst. The latter helps to avoid unnecessary emissions.

This results show that the microwave oxidation state analysis has the ability for transient applications on the road for TWC coated filters and honeycomb monoliths. Future work should focus on calibration and recalibration concepts with respect to catalyst aging. The influence of possible ash accumulation and the long-term stability of this technique should also be investigated.
